# Clinical Isolates of Shiga Toxin 1a–Producing *Shigella flexneri* with an Epidemiological Link to Recent Travel to Hispañiola

**DOI:** 10.3201/eid2010.140292

**Published:** 2014-10

**Authors:** Miranda D. Gray, Keith A. Lampel, Nancy A. Strockbine, Reinaldo E. Fernandez, Angela R. Melton-Celsa, Anthony T. Maurelli

**Affiliations:** Uniformed Services University of the Health Sciences, Bethesda, Maryland, USA (M.D. Gray, R.E. Fernandez, A.R. Melton-Celsa, A.T. Maurelli);; US Food and Drug Administration, College Park, Maryland, USA (K.A. Lampel);; Centers for Disease Control and Prevention, Atlanta, Georgia, USA (N.A. Strockbine)

**Keywords:** *Shigella*, Shiga toxin, bacteriophages, RecA, Hispañiola, bacteria

## Abstract

Shiga toxins (Stx) are cytotoxins involved in severe human intestinal disease. These toxins are commonly found in *Shigella dysenteriae* serotype 1 and Shiga-toxin–producing *Escherichia coli*; however, the toxin genes have been found in other *Shigella* species. We identified 26 *Shigella flexneri* serotype 2 strains isolated by public health laboratories in the United States during 2001–2013, which encode the Shiga toxin 1a gene (*stx_1a_*). These strains produced and released Stx1a as measured by cytotoxicity and neutralization assays using anti-Stx/Stx1a antiserum. The release of Stx1a into culture supernatants increased ≈100-fold after treatment with mitomycin C, suggesting that *stx_1a_* is carried by a bacteriophage. Infectious phage were found in culture supernatants and increased ≈1,000-fold with mitomycin C. Whole-genome sequencing of several isolates and PCR analyses of all strains confirmed that *stx_1a_* was carried by a lambdoid bacteriophage. Furthermore, all patients who reported foreign travel had recently been to Hispañiola, suggesting that emergence of these novel strains is associated with that region.

## Abstract 2

Production of Shiga toxin 1a in strains of *S. flexneri* has potential to cause serious clinical complications.

Shiga toxins (Stx) are potent AB_5_ cytotoxins that inhibit eukaryotic protein synthesis, eventually leading to host cell death (*1*). Infections with bacteria that produce Stx result in hemorrhagic colitis and can lead to serious complications like hemolytic uremic syndrome (HUS) (*2*). Although 4 species of *Shigella* cause bacillary dysentery, historically only *Shigella dysenteriae* type 1 has been recognized as carrying the genes for Shiga toxin (*stx*). Likewise, *S. dysenteriae* 1 is the only *Shigella* species that causes HUS as a complication of infection (*3*).

The genes encoding the toxin are found in an operon consisting of *stxA* and *stxB*. The *stx* locus in *S. dysenteriae* 1 is surrounded by DNA sequence homologous to lambdoid bacteriophage sequence; however, the toxin genes are not associated with a complete prophage genome (*4*,*5*). Insertion sequences flanking the *stx* region suggest that gene rearrangements occurred and resulted in a defective phage. As a consequence, viable phage are not recovered from *S. dysenteriae* 1 cultures even under conditions that induce phage production (*6*).

Stx has been extensively studied in Shiga toxin–producing *Escherichia coli* (STEC), notably *E. coli* O157:H7. STEC produce 2 variants of Stx: Stx1a (which differs from *S. dysenteriae* 1 Stx by 1 aa), and Stx2 (which shares 56% identity with Stx1a) (*7*,*8*). In contrast to the toxin genes in *S. dysenteriae* 1, those in STEC are generally carried by lambdoid prophages, which integrate into the host bacterial chromosome (*9*). The phage remains in a lysogenic state until environmental conditions induce expression of phage lytic cycle genes, leading to new phage production and lysis of the host bacterium. The *stx_1a_* and *stx_2_* loci are found within the late gene regions of the phage; therefore, induction of the lytic cycle increases expression of the toxin genes and host cell lysis allows for toxin release (*10*).

Recently, acquisition of *stx* genes in clinical isolates of other *Shigella* species has been reported (*11*,*12*). Three cases of infection with *S. dysenteriae* 4 were described, and all were shown to express *stx_1_*. No further characterization of the *stx_1_*-encoding *S. dysenteriae* 4 strains was reported; however, all 3 infected patients had reported recent travel to Hispañiola (*11*). An isolate of Shiga toxin–producing *S. sonnei* from a patient returning from the Ukraine was also characterized; the toxin genes were determined to be carried by a lambdoid prophage homologous to *stx*-encoding phages found in STEC (*13*).

We identified 26 clinical isolates of *S. flexneri* 2 that encode *stx_1a_*. DNA sequence and PCR analyses determined that *stx_1a_* is encoded by a lambdoid prophage. Characterization of the phage indicated that it behaves similarly to *stx*-encoding phages that infect STEC. Like the patients from whom *stx_1_*-encoding *S. dysenteriae* 4 was isolated, patients from whom *stx_1a_*-encoding *S. flexneri* 2 was isolated and who reported foreign travel had also recently visited Hispañiola. The potential consequences of an epidemiological link to this region are discussed.

## Methods

### Bacterial Strains and Growth Conditions

*Shigella* clinical isolates used in this study are listed in Table 1. *S. flexneri* strains were grown in Tryptic Soy Broth (BD Difco, Franklin Lakes, NJ, USA) at 37°C with aeration or on Tryptic Soy Broth plates containing 1.5% agar and 0.025% Congo red. *E. coli* K-12 strain MG1655 was grown in Luria-Bertani broth and on Luria-Bertani agar plates. Kanamycin and ampicillin were used at 50 μg/mL and 100 μg/mL, respectively.

**Table 1:** Isolation information for clinical strains of *Shigella flexneri* , USA, 2001–2013*

Strain	Source state (laboratory no.)	Isolation date	Recent travel destination
*stx_1a_-*positive			
BS937	HI (HI_N10–094)	2010 Mar	Haiti
BS938	MA (MA_12EN1615)	2012 May	NA
BS942	MA (MA_10EN1901)	2010 Sep	NR
BS943	MA (MA_11EN1036)	2011 Jun	NA
BS951	PA (05E00067)	2005 Jan	NR
BS954	PA (05E02261)	2005 Dec	Dominican Republic
BS955	PA (06E00134)	2006 Jan	NR
BS957	PA (06E00281)	2006 Feb	NR
BS958	PA (06E00283)	2006 Jan	NR
BS959	PA (06E00305)	2006 Feb	NR
BS960	PA (06E00941)	Unknown	Haiti
BS963	PA (08E01943)	2008 Sep	NR
BS965	PA (M09015890001A)	2009 Jul	NA
BS968	PA (M10005231001A)	2010 Feb	Haiti
BS971	PA (M11028960001A)	2011 Nov	Haiti
BS972	MA (12EN7814)	2012 Nov	NR‡
BS974	IN (01–3105)†	2001 Apr	Haiti
BS980	MA (05–3606)†	2005 Oct	NR‡
BS981	CT (06–3001)†	2005 Dec	Dominican Republic
BS982	GA (08–3370)†	2008 Apr	NR
BS988	CT (2012C-3273 †	2012 Jan	Haiti
BS989	CT (2013C-3310)†	2013 (month unknown)	NR
BS998	PA (M13004940001A)	2013 Mar	Haiti
BS999	MA (MA_13EN0428)	2013 Jan	Haiti
BS1010	MD (MDA10005139)	2009 Dec	Dominican Republic
BS1011	MD (MDA12018728)	2012 Jan	NA
*stx_1a_-*negative			
BS952	PA (05E00414)	2005 Apr	Peru
BS969	PA (M11015188001A)	2011 Jun	India
BS970	PA (M11015261001A)	2011 Jun	NR

*NA, no information available; NR, none reported; *stx_1a_*, Shiga toxin 1a gene. †Obtained by the Centers for Disease Control and Prevention. ‡No foreign travel reported, but patient had contact with a person who recently returned from Haiti.

### PCR Analysis of *stx_1a_*-Encoding *S. flexneri*

DNA lysates were used for PCR for *stx_1a_* with primers stx1-det-F1 and stx1-seq-R1, and for *stx_2_* with primers stx_F/stxR1. All strains were verified to contain the virulence plasmid of *S. flexneri* by PCR amplification of *virF* with primers VirF1 and VirF2.

To show that *stx_1a_* was phage encoded, we analyzed lysates by PCR with primer pairs Stx1R2/Phage_stxR2 and Phage_stx1F2/Stx1F2. The insertion site of the phage into *S. flexneri* locus S1742 was determined by amplifying the upstream region of S1742 and an early phage gene with primers S1742_up/Stx_phage_up and by amplifying a late phage gene and the downstream region of S1742 with primers Stx_phage_dn/S1742_dn. All PCRs were conducted by using PCR Master Mix (2X) (Fermentas, Pittsburgh, PA, USA) according to the manufacturer’s specifications. Primer sequences and expected amplicons are listed in Table 2.

**Table 2:** Primer pairs used for PCR analysis

Primer pair	Sequence, 5′ → 3′	Amplicon size, bp	Source
stx1-det-F1	GTACGGGGATGCAGATAAATCGC	698	(*14*)
stx1-seq-R1	GAAGAAGAGACTGAAGATTCCATCTG		
stx2_F4	GGCACTGTCTGAAACTGCTCCTGT	627	(*14*)
stx2R1	ATTAAACTGCACTTCAGCAAATCC		
VirF1	GCAAATACTTAGCTTGTTGCACAGAG	907	This study
VirF2	GGGCTTGATATTCCGATAAGTC		
VirB01	TTCTACCATCAATCTCCCTTCC	897	This study
IpaAFwd	GTATCTAGCGCCCTCAGCAAG		
IpaHF	GCGTTCCTTGACCGCCTTTCCGATACCG	628	This study
IpaHR	CTTTCAGCCGGTCAGCCACCCTCTGAGAG		
Stx1R2	AGCGAATGACATTCAGCGAATCTA	1,059	This study
Phage_stxR2	GACGCCATACAAGGAGTC		
Stx1F2	ACGCCTGATTGTGTAACTGGAAA	1,333	This study
Phage_stx1F2	CACTCGCGTCACTGTATG		
Stx_phage_up	GACCGCACACTGTGCTATC	1,155	This study
S1742 up	CCGTGCGGGTATTTAACAATAATGG		
Stx_phage_dn	AGTCAAACCGCGCTATTGG	1,224	This study
S1742 dn	TGCATGACAGAGGCAATAAACCCGAT		
RecAko-site1	GCTATCGACGAAAACAAACAGAAAGCGTTGGCGGCAGCACTGGGCCAGATTGTGTAGGCTGGAGCTGCTTC	1,609	This study
RecAko-site2	AAAATCTTCGTTAGTTTCTGCTACTCCTTCGCTGTCATCTACAGAGAAATCCATATGAATATCCTCCTTA		
RecA-1	ACATATTGACTATCCGGTATTACCCGG	1,148, 1,701*	This study
RecA-3	GACCGTCCGTGCACACATTATCTATT		

*Amplicon sizes for wild-type *recA* or insertion of *kan* cassette into *recA*, respectively.

### Construction of *recA* Mutants

*recA* was replaced with a kanamycin-resistance cassette by using λ red recombination (*15*). Primers RecAko-site1 and RecAko-site2 were used to amplify *kan* from pKD4 with 5′ and 3′ overhangs homologous to internal regions of *recA*. Kanamycin-resistant colonies of BS766 (*15*) were double purified and screened by PCR with primers RecA-1/RecA-3 for detection of the size difference between chromosomal *recA* and the kanamycin-resistance cassette. This mutant was used as the donor for growing a P1L4 lysate, which was used to transduce the *recA*::*kan* mutation into BS937, BS938, and BS974. Kanamycin-resistant transductants were purified and confirmed by use of PCR, as described above.

### Cytotoxicity Assay

The cytotoxicity of bacterial samples for Vero cells was determined as previously described (*16*,*17*). In brief, 100 μL of diluted samples was overlain in 96-well plates containing confluent monolayers of Vero cells and incubated for 48 hours at 37°C in 5% CO_2_. Viable cells were fixed with 10% formalin and stained with 0.13% crystal violet. The optical density (OD) of the stained wells was measured at 630 nm by using a BioTek (Winooski, VT, USA) EL800 spectrophotometric plate reader. The CD_50_ (cytotoxic dose that kills 50% of the cells) was calculated by determining the inverse dilution of the bacterial sample that was required to kill 50% of the Vero cells. 

### In Vitro Neutralization of Stx1a

Overnight supernatants were serially diluted 10-fold in medium. We mixed 100 μL of diluted samples with 100 μL medium, a 1:25 dilution of F45 polyclonal anti-Stx1/Stx1a antiserum (*17*,*18*), or a 1:25 dilution of rabbit polyclonal antiserum against *S. flexneri* whole cell lysate. Toxin samples were incubated with antibody for 2 hours at 37°C in 5% CO_2_. We then applied 100 μL of the toxin–antibody mixture to Vero cells and incubated as above.

### Mitomycin C Induction of Bacterial Lysis, Shiga Toxin 1a Production, and Prophage Induction

Overnight cultures of bacteria were inoculated 1:100 into Tryptic Soy Broth, and 2 hours after inoculation a final volume of 0.5 μg/mL mitomycin C (Sigma, St. Louis, MO, USA) was added to the cultures. To monitor the induction of bacterial lysis, we read the OD_600_ hourly over a period of 8 hours. Induction of bacterial lysis was noted as a 3-4–fold decrease in OD_600_ compared with the *stx_1a_*-negative control strains.

To determine the effect of mitomycin C on production of Stx1a and prophage induction, we collected whole cell lysates and supernatants 3 hours after addition of mitomycin C. Samples were then analyzed for cytotoxicity on Vero cells. For isolation of phage particles, supernatants were prepared similarly, except that a final concentration of 10 mmol/L MgSO_4_ and a drop of chloroform were added after centrifugation. As described previously, 100 μL of phage lysate was absorbed onto 200 μL of *E. coli* MG1655 for 20 minutes at 37°C (*19*). Molten L-agar top agar (*19*) containing 10 mmol/L MgSO_4_ was added to the phage/bacteria mixture and poured onto L-agar plates. Plates were incubated overnight at 37°C, and plaque-forming units (PFUs) were counted.

### Isolation of Lysogens

Supernatants containing phage were prepared from mitomycin C–induced culture of BS937. Phage lysate was spotted onto an L-soft agar overlay containing either *E. coli* MG1655 or *S. flexneri* 2457T and incubated overnight at 37°C. A loop from the zone of clearing was streaked for isolation of single colonies, which were subsequently screened for *stx_1a_* genes by PCR. Positive colonies from the initial screening were double colony purified, and PCR was repeated to ensure that the colonies were positive for *stx_1a_*. MG1655 lysogens were confirmed to not be contaminates of the donor strain, BS937, by testing for *S. flexneri* chromosomal and virulence plasmid genes by use of PCR primer pairs IpaHF/IpaHR and VirB01/IpaFWD (Table 2), respectively. Similarly, 2457T lysogens were analyzed by PCR as described above. Two independently isolated lysogens of MG1655 and 2457T were used for further analysis.

### Virulence Assays

Virulence-associated phenotypes were determined by a gentamicin protection invasion assay in HeLa cells and by plaque formation in L2 monolayers, as previously described (*20*,*21*). Both assays were conducted 3 independent times and included technical duplicates or triplicates in each individual experiment. 

### Whole-Genome Sequencing

DNA was isolated from overnight cultures by using a QIAGEN DNEasy Kit (Valencia, CA, USA). Samples were prepared for sequencing by using a Nextera XT DNA Sample Preparation Kit (Illumina, San Diego, CA, USA) and sequenced on an Illumina MiSeq sequencing system. The phage sequence was assembled by mapping the reads to the reference phage NC_004913.2 by using Bowtie 2 version 2.1.0 (http://sourceforge.net/projects/bowtie-bio/files/bowtie2/2.1.0/).

### Nucleotide Sequence Accession Number

The complete phage sequence of ϕPOC-J13 from strain BS937 was submitted to GenBank. The sequence is available under accession no. KJ603229.

## Results

### Identification and Epidemiology of *stx_1a_*-positive *S. flexneri* 2 Strains

BS937 and BS938 (Table 1) were acquired from the Hawaii and Massachusetts state laboratories, which had determined the isolates to be positive for *stx_1a_* by PCR. Both strains shared the same pulsed-field gel electrophoresis (PFGE) XbaI pattern, JZXX01.0357, as indicated in the Centers for Disease Control and Prevention PulseNet database (http://www.cdc.gov/pulsenet). To identify other clinical isolates of *S. flexneri* 2 that might be *stx_1a_*-positive, we searched the PulseNet database for strains that matched this PFGE pattern. From state public health laboratories, we obtained 18 additional strains of *S. flexneri* that matched this PFGE pattern. We also received time-matched, but not PFGE-matched, strains of *S. flexneri* as negative controls. Six additional strains that had been confirmed to be *stx_1a_*-positive *S. flexneri* were acquired from the Centers for Disease Control and Prevention. Clinical strains included in this study and their sources are listed in Table 1.

The *stx_1a_*-positive *S. flexneri* strains had been isolated over 13 years (2001–2013). They were isolated during all months except August, indicating that seasonality is not involved in the emergence and/or spread of *stx_1a_*-encoding *S. flexneri*. Among patients from whom *stx_1a_*-positive *S. flexneri* strains were isolated, no incidences of HUS were reported, suggesting that the *stx_1a_*-positive *S. flexneri* strains did not cause more severe disease than would typically be caused by such strains lacking *stx_1a_*. Among 22 patients for whom travel information was available, 9 reported no foreign travel or knowledge of contact with persons who had traveled. The 13 patients who reported travel had all recently visited Hispañiola or interacted with a traveler who had returned from this region (Table 1).

### Verification of Stx1a in *S. flexneri* Isolates

The presence of *stx_1a_* in the *S. flexneri* strains was confirmed by PCR (data not shown). All isolates that matched PFGE pattern JZXX01.0357 or had previously been shown to encode *stx_1a_* yielded a PCR product of the correct size for the toxin gene. PCR analysis for *stx_2_* did not produce a product. The 3 negative controls did not generate a PCR product for either *stx_1a_* or *stx_2_*.

**Figure 1:**
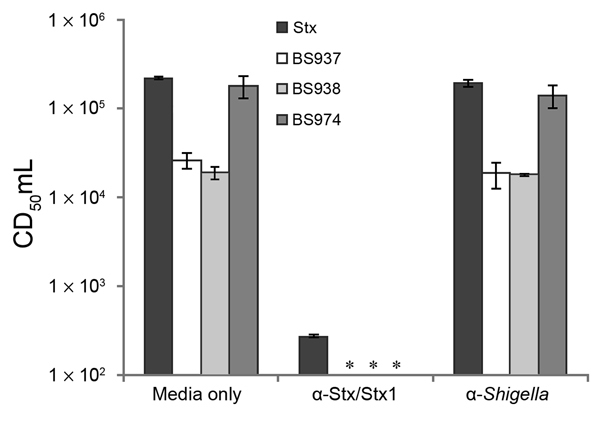
Shiga toxin 1a (Stx1a) is produced and released from *stx*_1a_–encoding *Shigella flexneri* isolates. Overnight supernatants from BS937, BS938, and BS974 were serially diluted 10-fold in medium alone, medium containing anti-Stx/Stx1a antiserum, or antiserum against whole cell lysates of *S. flexneri*. After 2 hours of incubation, samples were analyzed in a Vero cell cytotoxicity assay. Stx from *S. dysenteriae* 1 was included as a positive control. CD_50_/mL is defined as the reciprocal of the dilution of Stx1 that kills 50% of Vero cells. *CD_50_/mL was below the level of detection. Data are averages of 3 independent experiments. Error bars indicate standard error. α indicates anti.

To determine if Stx1a was released from the bacteria, we tested supernatants from overnight cultures in a Vero cell cytotoxicity assay. All *stx_1a_*-positive isolates released a toxin into the supernatant, which killed Vero cells. To confirm that the toxin responsible for killing the cells was Stx1a, we tested overnight supernatants from 3 representative isolates for Vero cell cytotoxicity after neutralization by anti-Stx/Stx1a antiserum. After neutralization, supernatants were no longer cytotoxic to Vero cells (Figure 1). These findings demonstrate that the extracellular product responsible for cytotoxicity to Vero cells is indeed Stx1a and not a different protein being released by the *stx_1a_*-expressing *S. flexneri*.

### Effects of Mitomycin C on Stx1a and Prophage Production 

*stx* is generally found encoded by functional prophages (*22*). The prophage lytic cycle can be induced with DNA damaging agents (*23*); therefore, to address whether the toxin carried in *stx_1a_*-encoding *S. flexneri* was associated with a prophage, we tested sensitivity to lysis when grown in the presence of mitomycin C. All Stx1a-producing *S. flexneri* isolates showed a sharp decrease in OD_600_ within 3–4 hours after addition of mitomycin C, whereas the *stx_1a_*-negative strains showed no decrease in OD_600_ (data not shown).

**Figure 2:**
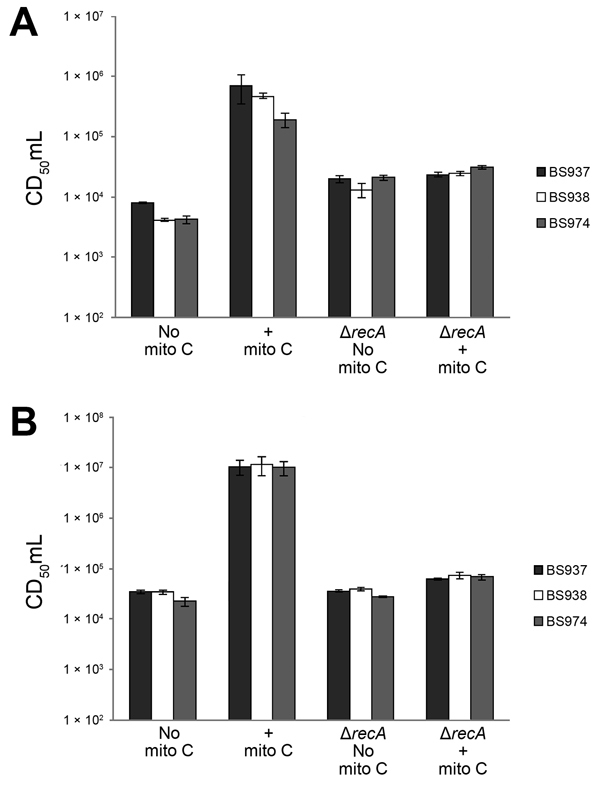
Mitomycin C induces production of Shiga toxin 1a (Stx1a) in a *recA*-dependent manner. Exponentially growing cultures of the indicated parental strains or *recA* mutants were grown with or without 0.5 μg/mL mitomycin C (mito C) for 3 hours. Supernatants (A) or whole cell lysates (B) were prepared for determination of cytotoxicity for Vero cells. CD_50_/mL values were determined as described in Figure 1. Data are averages of 3 biological replicates. Error bars indicate standard error. Δ indicates samples that are *recA* mutants.

**Figure 3:**
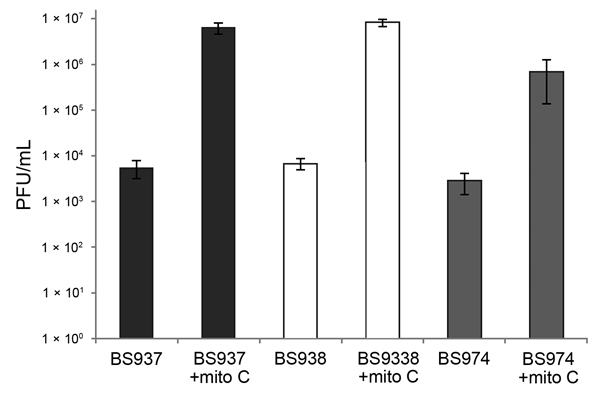
Infectious phage in the supernatants of Shiga toxin 1a gene (*stx_1a_*)–encoding *Shigella flexneri* are induced with mitomycin C (mito C) treatment. Supernatants were collected from exponential cultures of BS937, BS938, and BS974 grown with or without 0.5 μg/mL mito C for 3 h. The number of infectious phage particles was determined by a soft agar overlay method that used *Escherichia coli* MG1655 as the recipient. Plaque forming units (PFUs) of phage lysate were counted after 24 h incubation. Data are averages of 3 independent experiments. Error bars indicate standard error.

Because all *stx_1a_*-encoding *S. flexneri* isolates behaved similarly in the assays, we selected 3 isolates (BS937, BS938, and BS974) to characterize more thoroughly. To further investigate the response to mitomycin C, we grew log-phase cultures and collected samples to measure cell-associated and released toxin. Supernatants and whole cell lysates from bacteria treated with mitomycin C exhibited elevated cytotoxicity to Vero cells (Figure 2). To determine if Stx1a–producing *S. flexneri* generated infectious phage, we analyzed supernatants from untreated and mitomycin C–treated cultures in a PFU assay. After treatment with mitomycin C, PFUs in supernatants of BS937, BS938, and BS974 increased ≈1,000-fold (Figure 3).

Induction of the prophage lytic cycle by mitomycin C is caused by the SOS response (*24*). During the SOS response, the bacterial protease RecA becomes active and cleaves the phage repressor *c*I, which maintains the phage in a quiescent state under non-SOS conditions. Cleavage of *c*I enables transcription of the phage antiterminator Q, which activates the late phage genes, including *stx* (*25*). STEC *recA* mutants no longer release toxin or respond to agents that trigger the SOS response (*24*,*26*,*27*).

To investigate whether similar regulation occurs in Stx1a-producing *S. flexneri*, we constructed *recA* deletions in BS937, BS938, and BS974. *recA* mutants were cultured with mitomycin C as above, and samples were collected to measure the presence of toxin and phage. In the absence of mitomycin C, the *recA* mutants produced and released toxin in amounts comparable to those of the parental strains; however, when cultured with mitomycin C, the *recA* mutants did not exhibit increased cytotoxicity to Vero cells (Figure 2). Additionally, in the absence or presence of mitomycin C, no PFUs were enumerated from the *recA* mutants. Collectively, these data suggest that *stx_1a_* in *S. flexneri* is carried by a lambdoid prophage.

### *stx_1a_* Carriage by a Lambdoid Prophage in *S. flexneri*

**Figure 4:**
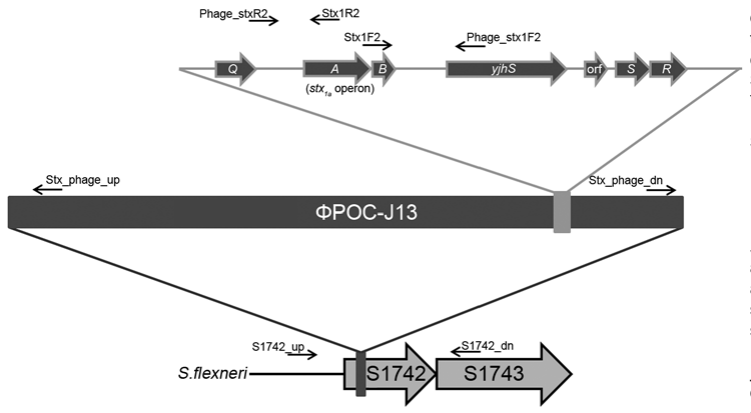
Schematic of PCR designed to determine that Shiga toxin 1a gene (*stx_1a_*) is phage encoded and inserted into the S1742 locus of *Shigella flexneri*. The genetic map shows the insertion of ϕPOC-J13 into locus S1742 of *stx_1a_*-encoding *S. flexneri*. Location and direction of the primers used for PCR analyses are indicated. The top part of the figure indicates the genes flanking the *stx1a* operon. Q, antiterminator; A and B, Stx1a subunits that form the assembled toxin (A is the *stx1aA* subunit and B is the *stx1aB* subunit); *yshS*, a hypothetical protein that shares homology with *yjhS* from *Escherichia coli* K-12; orf, open reading frame; *S*, gene that encodes the phage holin; *R*, gene that encodes the phage endolysin.

To identify the location of *stx_1a_*, we sequenced BS937, BS938, and BS974. Whole-genome sequencing confirmed that *stx_1a_* was encoded within a 62-kb lambdoid prophage. To extend the analysis to all the clinical isolates in this study, we used the PCR strategy and primer design shown in Figure 4. To ensure that *stx_1a_* in all isolates was phage encoded, we designed primers to amplify from *stxA_1a_* and *stxB_1a_* (encoding subunits A and B of Stx1a) and 1 kb either upstream or downstream of the *stx_1a_* operon. All *stx_1a_*-positive strains yielded a PCR product consistent with the toxin being phage encoded. No DNA was amplified from the *stx_1a_*-negative isolates.

**Figure 5:**
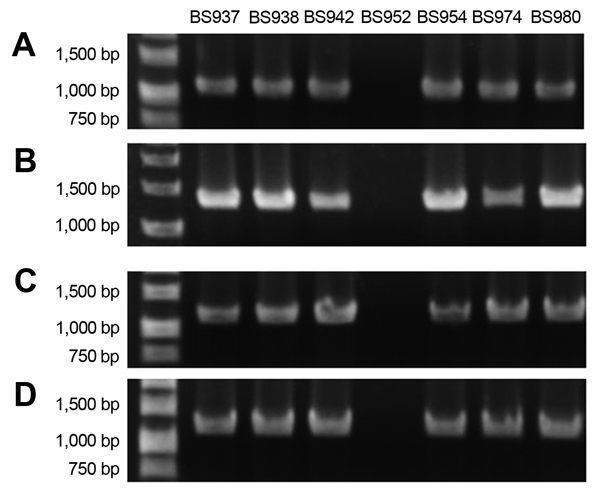
PCR results from representative clinical isolates illustrate that the Shiga toxin 1a gene (*stx_1a_*) is phage encoded and inserted into the S1742 locus of *Shigella flexneri*. PCRs based on the primer scheme detailed in Figure 4 are shown for 6 *stx_1a_*-positive strains (BS937, BS938, BS942, BS954, BS974, BS980) and 1 *stx_1a_*-negative isolate (BS952). To show that *stx_1a_* is phage encoded, we used primer pairs Stx1R2/Phage_stxR2 (A) and Phage_stx1F2.Stx1F2 (B). To analyze the insertion of ϕPOC-J13 into locus S1742, we used primer pairs S1742_up/Stx_phage_up (C) and Stx_phage_dn/S1742_dn (D).

Whole-genome sequencing revealed that the phage was inserted into locus S1742 (which encodes a putative oxidoreductase) of the *S. flexneri* chromosome. Primers were designed (Figure 4) to determine if the *stx_1a_*-encoding phage inserted into S1742 for all isolates. All *stx_1a_*-positive strains generated the expected PCR product when amplified with primers specific for the early phage sequence and upstream of S1742 and with primers directed to the late phage sequence and downstream of S1742. None of the *stx_1a_*-negative strains yielded an amplified product with the primer pairs. A representative gel of the 4 amplifications for 1 *stx_1a_*-negative and 6 *stx_1a_*-positive isolates is shown in Figure 5. These data suggest that a phage has integrated into all *stx_1a_*-positive isolates. We named this *stx_1a_*-encoding phage ϕPOC-J13.

### Lysogeny of Laboratory Strains of *E. coli* and *S. flexneri* with ϕPOC-J13

*E. coli* MG1655 and *S. flexneri* 2457T were lysogenized as described earlier. To test for production and release of Stx1a, we examined whole cell lysates and supernatants from overnight cultures of MG1655 and 2457T lysogens in Vero cell cytotoxicity assays. The average CD_50_/mL of supernatants from MG1655 and 2457T lysogens was 1 ×10^5^, similar to that of the *stx_1a_*-positive *S. flexneri* clinical isolates. Lysogens were also tested for the presence of phage in supernatants of overnight cultures by determining PFUs. The 2457T lysogens released ≈10^6^ PFUs/mL; however, no viable phage could be recovered from MG1655 lysogens.

To confirm that *stx_1a_* in the lysogens was phage associated, we conducted PCR amplification as described above (Figure 4). The MG1655 and 2457T lysogens yielded a PCR product that indicated that the phage regions upstream and downstream of *stx_1a_* were present. Lysogens were also tested for integration of the phage into locus S1742 by use of the primers illustrated in Figure 4. In 2457T lysogens, ϕPOC-J13 inserted into S1742 and produced a PCR product of the expected size. Similarly, in lysogens of MG1655, ϕPOC-J13 inserted into *ynfG*, the *E. coli* S1742 homologue. These findings demonstrate that the integration of ϕPOC-J13 is site specific, as has been shown for other lambdoid prophages (*9*).

### Virulence Phenotypes of Shiga Toxin 1a–producing *S. flexneri*

We wanted to determine if the presence of ϕPOC-J13 altered the virulence phenotypes associated with *S. flexneri*. Invasion and plaquing efficiencies of BS937, BS938, and BS974 were compared with those of laboratory strain 2457T (Table 3). BS938 and BS974 exhibited similar invasion efficiency as 2457T; however, invasion with BS937 was significantly higher. All 3 Stx1a–producing *S. flexneri* isolates showed plaquing efficiency comparable to that of 2457T, and the plaque diameters were consistent among all strains. Although it is unclear why BS937 was more invasive, the comparable level of cell-to-cell spread suggests that ϕPOC-J13 does not appreciably alter the virulence properties of *S. flexneri*.

**Table 3:** Virulence properties of *Shigella flexneri* strains

Strain	Invasion, % ± SE*	Plaquing efficiency, % ±SE†
2457T	0.35 ± 0.04	4.34 ± 0.63
BS937	1.24 ± 0.27	3.22 ± 0.56
BS938	0.22 ± 0.03	4.34 ± 0.79
BS974	0.40 ± 0.09	3.24 ± 0.44

*Number of colony-forming units (CFUs) recovered from HeLa cells after gentamicin protection divided by the input CFUs. †Number of plaques formed on L2 monolayers divided by the input CFUs.

## Discussion

Bacteriophages are recognized for their contribution to the genetic diversity of bacteria and for their capacity to transfer virulence factors (*28*). It was first noted in the early 1980s that *stx* in *E. coli* was encoded by a lambdoid bacteriophage (*29*,*30*). We have identified a new *stx_1a_*-encoding bacteriophage, ϕPOC-J13, from clinical isolates of *S. flexneri*. Generally, the acquisition of toxin genes is thought to increase the virulence of a bacterial species. However, according to the available clinical data and our in vitro virulence assays, the production of Stx1a in *S. flexneri* does not seem to increase pathogenicity within the host.

Characterization of ϕPOC-J13 determined that it behaves similarly to *stx*-encoding phages found in STEC; however, some differences are notable. First, although ϕPOC-J13 responded to DNA damaging treatment by inducing the lytic cycle and induction was RecA-dependent, *recA* mutants of *S. flexneri* Stx1a–producing strains still maintained a level of Stx1a production and release comparable to that of the noninduced parental strains. In contrast, *recA* mutants of STEC produce a very low level of Stx, and the toxin that is present remains largely cell associated rather than being released into the supernatant (*24*,*26*,*27*). One explanation for the differences in phenotype between Stx1a-producing *S. flexneri* *recA* mutants and the STEC mutants might be that all STEC *recA* mutants examined encoded *stx_2_*; thus, the regulation of *stx_1a_*- and *stx_2_*-encoding phages might vary. Additionally, although Stx1a was produced in *S. flexneri recA* mutants, viable phage particles were not recovered. *stx_1a_* has an upstream promoter that is not dependent on induction of the phage lytic cycle (*31*). A similar promoter might be responsible for the baseline level of Stx1a produced in the *S. flexneri recA* mutants and would explain the lack of infectious phage in the mutants.

ϕPOC-J13 lysogenized laboratory strains of *E. coli* and *S. flexneri*. Viable phage particles were recovered from the supernatants of 2457T lysogens but not from those of MG1655 lysogens, even though Stx1a was produced and released by lysogens of both species. This result might suggest that the stability and/or assembly of ϕPOC-J13 varies according to the host bacterium. Host differences in the regulation of ϕPOC-J13 might also account for the discrepancies between the *recA* mutants of Stx1a-producing *S. flexneri* and STEC. Our future studies will compare the differences in regulation of *stx_1a_* in ϕPOC-J13 with that of known STEC phages. 

Another aspect of these *stx_1a_*-encoding *S. flexneri* isolates is their potential epidemiological link to Hispañiola. Although some patients reported no travel, ≈60% reported travel to this region or interaction with a traveler returning from this region. Most of our clinical isolates came from public health laboratories in the eastern United States, suggesting a possible focus in that area. However, the large number of isolates from the eastern United States might simply reflect the large Haitian immigrant population in this region and the resultant frequent travel to Haiti (*32*). Nevertheless, further surveillance of Stx1a–producing *S. flexneri* is warranted to determine the extent of their emergence in Hispañiola.

The epidemiological link to Hispañiola generates many questions about what has led to the emergence of these strains. The earliest Stx1a–producing *S. flexneri* isolates pre-date the earthquake that struck Haiti in January 2010. Thus, this natural disaster is not linked to the presence of *stx_1a_*-encoding *S. flexneri* in the region. One possibility is that the ecosystem in Hispañiola is favorable for the acquisition of ϕPOC-J13 by *S. flexneri* and possibly other *Shigella* species. An environmental reservoir of *Shigella* spp. has never been identified; therefore, it is tempting to speculate that production of Stx1a might give *S. flexneri* a survival advantage in the aquatic environment. In accordance with this hypothesis, studies on the survival of *Shigella* spp. in amebae indicate that *S. dysenteriae* 1 can persist longer than *S. flexneri* within *Acanthamoeba castellanii* (*33*,*34*). In addition, Stx–producing bacteria can kill the protozoan *Tetrahymena thermophile* to avoid consumption by this predator (*35*). Thus, Stx1a might benefit *S. flexneri* by providing a defense against eukaryotic predators.

It will be important to study clinical isolates of other *Shigella* species and bacterial genera to determine whether they also harbor ϕPOC-J13; we expect that the occurrence of this *stx_1a_*-encoding phage will be more widespread. Although toxin production in *S. flexneri* did not suggest an increase in pathogenicity, the consequences of the emergence of such Stx1a–producing strains are impossible to predict. Future studies that address these questions will provide a better understanding of the emergence of *stx_1a_*-encoding *S. flexneri*.
